# Granulicatella elegans Infective Endocarditis in a Pediatric Patient

**DOI:** 10.7759/cureus.111917

**Published:** 2026-07-01

**Authors:** Marta Valerio, Margarida Caldeira, Maria Bandeira Duarte, Rui Anjos

**Affiliations:** 1 Paediatric Department, The Unidade Local de Saúde da Lezíria (ULS Lezíria), Santarém, PRT; 2 Paediatric Cardiology Department, The Unidade Local de Saúde (ULS) Lisboa Ocidental, Hospital de Santa Cruz, Lisbon, PRT

**Keywords:** congenital heart disease, culture-negative endocarditis, granulicatella elegans, infective endocarditis, nutritionally variant streptococci

## Abstract

*Granulicatella elegans (G. elegans)* is a fastidious, nutritionally variant streptococcus (NVS) that represents a rare but clinically significant cause of infective endocarditis (IE), particularly in patients with congenital heart disease (CHD). Diagnosis is frequently delayed owing to its specific culture requirements and an indolent, non-specific clinical presentation. We present a case of a six-year-old male with complex CHD who presented with an 11-day history of low-grade fever, anorexia, and asthenia. Seven months prior, he had undergone dental caries repair without antibiotic prophylaxis. Transthoracic echocardiography (TTE) showed no vegetations. After 18 days of incubation in enriched culture media, blood cultures yielded the identification of *G. elegans*. A diagnosis of possible IE was established per the modified Duke criteria. Treatment with ceftriaxone and gentamicin was initiated per the European Society of Cardiology (ESC) 2023 guidelines, subsequently switched to vancomycin due to hematological toxicity. The patient was discharged in stable condition and remained asymptomatic at a three-year follow-up. Comparison with five previously published pediatric cases of Granulicatella IE was made. *G. elegans* IE should be considered in any child with CHD and prolonged unexplained fever, even in the absence of classical echocardiographic findings.

## Introduction

Infective endocarditis (IE) in the pediatric population is a rare but potentially life-threatening condition, with an estimated incidence below five per 100,000 children per year [1-2]. Congenital heart disease (CHD) is the predominant predisposing factor, accounting for 50-70% of cases, and the most common causative organisms are *Staphylococcus aureus* and viridans group streptococci [1-2]. *Granulicatella elegans (G. elegans)* belongs to the group of nutritionally variant streptococci (NVS), which also includes *G. adiacens, G. balaenopterae*, and *Abiotrophia defectiva*. As a group, NVS are responsible for approximately 5-6% of all IE cases [3-5]. *G. elegans* is the most fastidious among them, accounting for roughly 8% of NVS-related IE, which represents approximately 0.48% of all IE cases [3,6-7]. Its growth requires pyridoxal or L-cysteine supplementation in culture media, contributing to prolonged incubation times, frequent negative standard blood cultures, and diagnostic delay [4,8].

The clinical presentation is characteristically subacute and indolent (including low-grade fever, fatigue, and anorexia) with only mild elevation of inflammatory markers, further obscuring the diagnosis. The indolent course and frequently negative blood cultures of *G. elegans* IE place it within the broad differential of culture-negative endocardial lesions. The principal non-infective mimic is nonbacterial thrombotic endocarditis (NBTE, or marantic endocarditis), characterized by sterile fibrin-platelet vegetations on cardiac valves in the setting of a hypercoagulable state, most commonly advanced malignancy or autoimmune disease [9]. Although NBTE is predominantly an adult entity and exceptionally rare in children, its consideration reinforces the need to exclude non-infective causes of valvular vegetations and persistently negative cultures before a diagnosis of IE is established.

NVS are constituents of the normal oral microflora and are frequently found in dental plaque. Invasive dental procedures are a recognized mechanism for transient bacteremia and subsequent endocardial seeding. Current guidelines recommend antibiotic prophylaxis before high-risk dental procedures in patients at highest risk for adverse IE outcomes, including those with CHD and prior cardiac surgery [10]. Published pediatric cases of *Granulicatella* IE are extremely rare. We present a case of a child with complex CHD, with particular attention to a dental procedure performed without antibiotic prophylaxis as a possible contributing portal of entry, and compare it with previously published pediatric cases.

## Case presentation

A six-year-old male was admitted to the emergency department (ED) with an 11-day history of low-grade nocturnal fever (maximum axillary temperature of 38°C, responsive to antipyretics), anorexia, asthenia, and occasional dry cough. No chest pain, palpitations, or respiratory, gastrointestinal, or urinary symptoms were reported. His medical history was significant for complex CHD, including a large ventricular septal defect (VSD), patent foramen ovale, ostium secundum atrial septal defect, patent ductus arteriosus, and coarctation of the aorta with a hypoplastic aortic valve. In the neonatal period, he had undergone pulmonary artery banding and surgical correction of the aortic coarctation. He remained clinically stable under regular pediatric cardiology follow-up. Although surgical correction of the VSD had been proposed, it had not yet been performed. He was not taking any regular medications, and routine immunizations were up to date.

Seven months before admission, the patient had undergone dental caries repair at a general dental practice. Despite the patient’s documented complex CHD and prior cardiac surgery, placing him clearly within the highest-risk category for IE according to current guidelines [10], no prophylaxis was recorded. No other potential portal of entry was identified from the history. The patient denied recent travel, the consumption of unpasteurized dairy products or untreated water, and there was no history of recent venous access or instrumentation. It should be noted that the seven-month interval between the procedure and presentation precludes definitive attribution of causality, and the possibility of other unidentified sources of bacteremia cannot be excluded.

On admission, the patient was hemodynamically stable with normal vital signs. A previously documented grade 4/6 systolic murmur was audible at the left sternal border, consistent with the known VSD. Peripheral pulses were symmetrical and normal. No hepatosplenomegaly was detected. A systematic search for peripheral stigmata of IE, including splinter hemorrhages, Osler nodes, Janeway lesions, and Roth spots, was negative. No rash or features of Kawasaki disease were present. Initial laboratory investigations showed a mild inflammatory response: leukocyte count of 9,200/μL (normal differential), C-reactive protein (CRP) of 5.72 mg/dL, and erythrocyte sedimentation rate (ESR) of 59 mm/hour. Chest radiography and abdominal ultrasound were unremarkable. Renal and hepatic function tests were normal.

**Figure 1:**
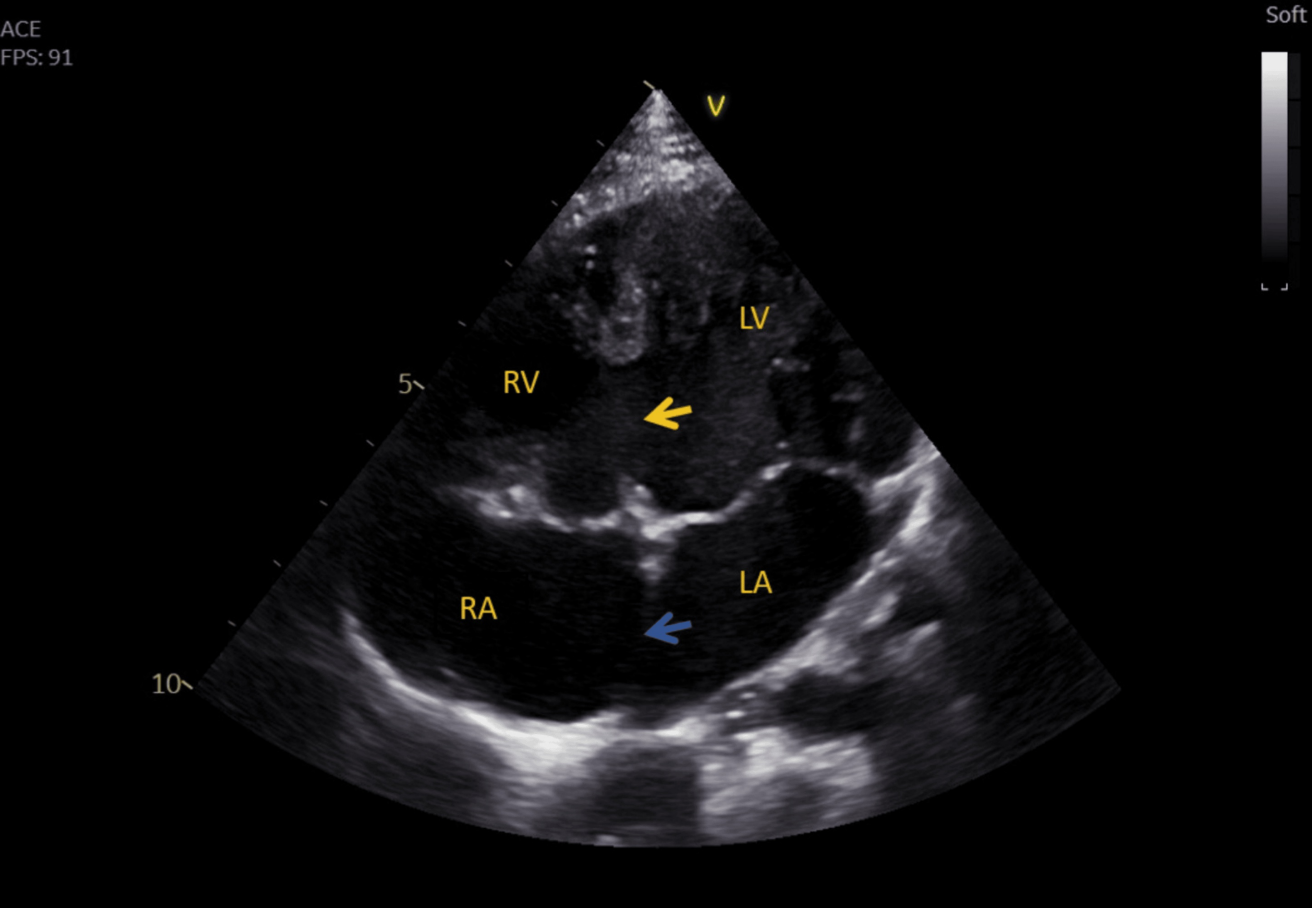
Transthoracic echocardiogram, apical four-chamber view Left ventricle is slightly dilated; right ventricle is small. Yellow arrow: large interventricular septum defect; blue arrow: ostium secundum interatrial septum defect; RA: right atrium; LA: left atrium; RV: right ventricle; LV: left ventricle

**Figure 2:**
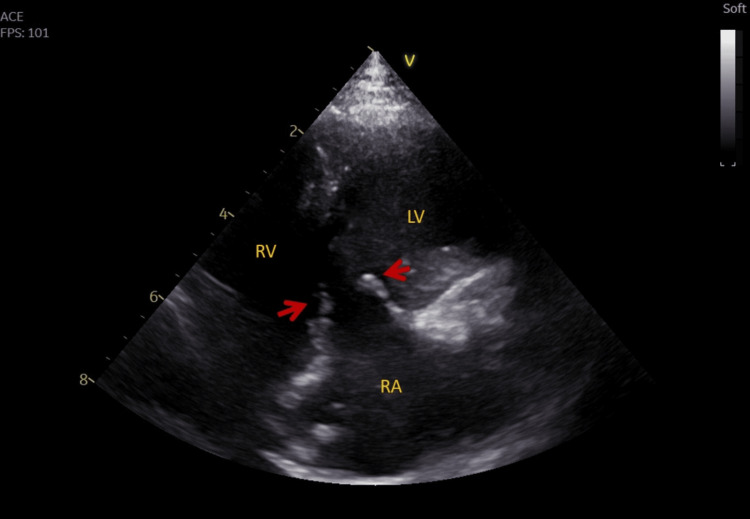
Transthoracic echocardiogram, modified parasternal long axis to see the tricuspid valve Red arrow: tricuspid valve leaflets RA: right atrium; RV: right ventricle; LV: left ventricle

Due to persistent fever of unknown origin, the patient was admitted for further investigation. A peripheral blood culture was obtained. He remained febrile during the initial days of hospitalization, so empirical therapy with ceftriaxone and metronidazole was initiated to provide coverage, including against anaerobic organisms. Transthoracic echocardiography (TTE) showed findings consistent with the patient’s documented baseline anatomy, with no evidence of vegetations, valve thickening, perivalvular abscess, or new regurgitation (Figures 1, 2).

The patient showed clinical improvement, with resolution of fever and normalization of inflammatory markers five days after the initiation of antibiotic therapy. Blood culture was positive for a Gram-positive bacterium displaying morphological pleomorphism on Gram staining, appearing as bacilli in the initial nutrient-depleted environment and raising clinical suspicion of a fastidious organism. Samples were referred to a specialized reference microbiology laboratory for incubation in pyridoxal-supplemented enriched media. After 18 days, *G. elegans* was identified. Antibiotic susceptibility testing could not be performed, as the reference laboratory had no validated, standardized method for this fastidious organism. Subsequent blood cultures were sterile.

Based on the modified Duke criteria [11], a diagnosis of possible infective endocarditis was established: three minor criteria were fulfilled: (1) fever ≥ 38°C; (2) predisposing cardiac condition (complex CHD with prior cardiac surgery); and (3) positive blood culture for a recognized IE-associated organism not meeting major criterion threshold. No major criteria were fulfilled: echocardiography showed no vegetations, and blood cultures did not grow a typical major-criterion organism. Antibiotic therapy was adjusted per the European Society of Cardiology (ESC) 2023 guidelines for *Granulicatella* IE [10]: ceftriaxone for four weeks combined with gentamicin for the first two weeks. The patient responded favorably, maintaining apyrexia with normalization of inflammatory markers. Repeated TTE and transesophageal echocardiography (TEE) showed no new findings and no vegetations.

On day 22, hematological monitoring revealed leukopenia (leukocytes 2,100/μL), neutropenia (neutrophils 500/μL), and thrombocytopenia (platelets 92,000/μL), consistent with a drug-related adverse reaction. Both ceftriaxone and gentamicin were discontinued, and therapy was switched to vancomycin for an additional 14 days. Blood counts normalized within one week. In total, the patient received 11 days of gentamicin, 22 days of ceftriaxone, and 14 days of vancomycin. Laboratory investigations performed throughout hospitalization are summarized in Table 1.

**Table 1:** Laboratory investigations during hospitalization Clinically relevant laboratory investigations performed during hospitalization are presented. Day 5 corresponds to five days after initiation of antibiotic therapy. Day 22 corresponds to the onset of hematological toxicity CRP: C-reactive protein; ESR: erythrocyte sedimentation rate

Parameters	Patient values (at admission)	Patient values (5th day)	Patient values (22nd day)	Reference values
Leukocyte count (/μL)	9,200	5,600	2,100	5,000–15,000
Neutrophils count (/μL)	6,600	3,090	500	1,500-8,000
Platelet count (/μL)	207,000	283,000	92,000	150,000–400,000
CRP (mg/dL)	5.72	0.95	0.12	< 0.5
ESR (mm/hour)	59	35	No data	< 20

The patient was discharged in stable clinical condition. At three-year follow-up, he remained fully asymptomatic, with normal serial echocardiography, sterile blood cultures, and no evidence of recurrence or late complications.

## Discussion

We discuss a rare case of *G. elegans* IE in a six-year-old male with complex CHD, a possible dental portal of entry in the absence of antibiotic prophylaxis, and a clinically challenging presentation characterized by prolonged low-grade fever, negative echocardiography, delayed microbiological identification, and antibiotic toxicity - yet a favorable long-term outcome. Comparison with five previously published pediatric cases (Table 2) reveals a remarkably consistent clinical phenotype across this rare entity.

**Table 2:** Comparison with published pediatric cases of Granulicatella spp. IE Surgical treatment refers to surgery directed at IE (e.g., valve intervention). Elective surgical correction of the underlying congenital defect, unrelated to the infective episode, was undertaken during follow-up in the case reported by Albano et al. and was planned but not yet performed in the present case IE: infective endocarditis; CHD: congenital heart disease; VSD: ventricular septal defect; PDA: patent ductus arteriosus; ASD: atrial septal defect; PFO: patent foramen ovale; CoAo: coarctation of the aorta; AoV: aortic valve

Study	Patient age (years)	Sex	Underlying CHD	Species	Echocardiography	Antibiotic regimen	Surgical treatment for IE
De Luca et al., 2013 [8] — case 1	7	Male	Shone syndrome + pulmonary valve homograft	G. adiacens	Positive (vegetation)	Meropenem + penicillin	No
De Luca et al., 2013 [8] — case 2	5	Female	Post-cardiac catheterization	G. adiacens	Negative	Meropenem + antistreptococcal therapy	No
Holloway et al., 2021 [3]	9	Male	Pulmonary atresia + Ebstein’s anomaly	G. elegans	Negative	Ceftriaxone + gentamicin → amoxicillin	No
Granda-Jiménez et al., 2023 [4]	8	Female	Patent ductus arteriosus	G. elegans	Positive (vegetation)	Ceftriaxone + gentamicin	No
Albano et al., 2022 [5]	13	Male	Perimembranous VSD	G. elegans	Positive (vegetation)	Ceftriaxone + gentamicin	No (elective VSD closure during follow-up, unrelated to IE)
Present case, 2025	6	Male	Complex CHD: VSD + PFO + ASD + PDA + CoAo + hypoplastic AoV	G. elegans	Negative	Ceftriaxone + gentamicin → vancomycin (hematological toxicity)	No (elective VSD correction planned, unrelated to IE)

CHD was present in all six published pediatric cases, representing a prevalence higher than the 50-70% reported for pediatric IE in general [1-2]. The underlying defects were diverse, suggesting that the nature of the structural lesion is less determinant than the hemodynamic turbulence it generates, which promotes endocardial damage and facilitates bacterial adhesion. A dental caries repair performed seven months before admission represents a possible portal of entry, though a definitive causal link cannot be established given the prolonged interval and the absence of microbiological confirmation. *Granulicatella* species are frequent constituents of the normal oral flora and dental plaque, and caries repair is known to produce transient oral bacteremia [5,12].

The seven-month interval does not exclude this pathway, as *G. elegans *produces a characteristically slow, subacute infection in which weeks to months may elapse between endocardial seeding and overt illness [3,8]. Other sources of bacteremia cannot be excluded. Notably, no antibiotic prophylaxis was administered before the dental procedure, despite the patient’s complex CHD and prior cardiac surgery representing a high-risk profile per ESC 2023 guidelines [10]. This case highlights the importance of systematic communication between cardiologists and dental practitioners regarding prophylaxis indications in high-risk pediatric patients.

Echocardiographic vegetations were present in only three of the six cases (50%): De Luca et al. [8] case one, and two further published cases [4-5]. In the remaining three - De Luca et al. [8] case two, one further published case [3], and the present case - echocardiography was repeatedly negative despite a clinically and microbiologically supported diagnosis of IE in all three. This finding, observed in three of six cases, may reflect genuinely small or absent vegetations attributable to the subacute infection pattern of *Granulicatella*, or the known limitations of echocardiographic resolution in structurally complex pediatric hearts. As a consequence, the modified Duke criteria [11] classified half of the cases - including the present one - as possible rather than definite IE. This argues strongly that echocardiographic negativity should not delay IE-directed treatment in a child with CHD and persistent bacteremia caused by a recognized IE-associated organism.

Delayed microbiological identification is a defining feature of *Granulicatella* IE. In the two cases with documented time to identification - one published case (21 days) [3] and the present case (18 days) - the time to identification exceeded standard blood culture reporting windows, raising the risk of misclassification as culture-negative endocarditis. This delay results directly from the organism’s pyridoxal dependence: sub-passaging onto conventional blood agar without supplementation typically fails, leading *Granulicatella* to be misidentified as a non-viable Gram-positive organism or overlooked entirely. Referral to specialized microbiology laboratories with enriched media capability is therefore essential in any child with CHD and unexplained bacteremia. Antibiotic susceptibility testing was performed in only two of six cases (33%).

In one published case [5], the antibiogram revealed resistance to macrolides, underscoring the therapeutic implications of achieving susceptibility testing whenever possible. The inability to perform susceptibility testing in the present case meant antibiotic choices were entirely empirical, a limitation acknowledged given the known variability in *Granulicatella* susceptibility to beta-lactams and aminoglycosides [12]. *Granulicatella* spp. require pyridoxal- or L-cysteine-supplemented media to grow, and standard automated and disc-diffusion systems are not validated for nutritionally variant streptococci; in the absence of established methodology and interpretive breakpoints, reliable in vitro susceptibility results frequently cannot be obtained even in specialized reference laboratories [12].

Ceftriaxone combined with gentamicin was the most frequently used definitive regimen (five of six cases), consistent with ESC 2023 recommendations [10]. Treatment duration ranged from four to six weeks across all cases. All six patients achieved clinical cure without requiring surgical valve intervention, representing a strikingly more favorable outcome than that reported in adult Granulicatella IE series, in which surgical intervention is required in approximately 25 to 30% of cases and mortality reaches 17 to 20% [6-7,13]. Notably, when cardiac surgery was performed, it addressed the underlying congenital defect rather than the endocarditis itself. In the case reported by Albano et al., the pre-existing VSD was electively closed during follow-up [5], and in the present case, elective surgical correction of the VSD had likewise been planned independently of the infective episode. In no case was surgery required to treat the endocarditis. Whether this reflects a genuinely better pediatric prognosis, earlier specialist referral, or publication bias toward favorable outcomes cannot be determined from this small series.

Antibiotic regimen modification was required in two of six cases (33%): in one published case [8], treatment failure necessitated escalation to meropenem; in the present case, hematological toxicity - leukopenia, neutropenia, and thrombocytopenia on day 22 - required discontinuation of both ceftriaxone and gentamicin, with transition to vancomycin. This complication, consistent with a drug-related adverse reaction in the context of prolonged combination antibiotic therapy, underscores the need for systematic hematological monitoring throughout the treatment course in pediatric patients on extended antibiotic regimens. The three-year recurrence-free follow-up documented in the present case provides reassurance that antibiotic therapy alone is curative even in complex CHD without echocardiographic vegetations.

To our knowledge, this is among the few pediatric cases of *G. elegans* IE to combine repeatedly negative echocardiography, a markedly delayed microbiological identification (18 days), and significant antibiotic-related hematological toxicity necessitating a change of regimen, while achieving cure with antibiotic therapy alone and one of the longest recurrence-free follow-up periods reported to date (three years). Beyond the individual case, this report consolidates all six published pediatric cases into a single comparative analysis, delineating a consistent clinical phenotype that may assist clinicians in recognizing this rare entity.

This report has several inherent limitations. The presumed portal of entry was not confirmed microbiologically, and the seven-month interval between the dental procedure and presentation precludes establishing a definitive causal relationship. Furthermore, the diagnosis was classified as possible rather than definite infective endocarditis according to the modified Duke criteria, owing to the absence of echocardiographic vegetations. Additionally, antibiotic susceptibility testing could not be performed, necessitating empiric antimicrobial therapy throughout the course of treatment.

## Conclusions

*G. elegans *IE in children presents a consistent and diagnostically challenging phenotype: pediatric patients with CHD, prolonged low-grade fever, absent peripheral stigmata, frequent absence of echocardiographic vegetations, and substantially delayed microbiological identification. Comparison across all six published pediatric cases confirms this pattern and demonstrates that antibiotic therapy alone is curative, with no requirement for surgical intervention and no mortality reported in published cases. Two key lessons emerge from this case and the comparative literature. First, IE caused by fastidious organisms must be actively considered in any child with CHD and prolonged unexplained fever or bacteremia, irrespective of echocardiographic findings - prompting blood cultures in enriched media with extended incubation and referral to specialized microbiology laboratories where necessary. Second, antibiotic prophylaxis guidelines for high-risk dental procedures in children with CHD must be consistently applied at the point of dental care, as the present case highlights the importance of this measure in high-risk pediatric patients. The pathogenic role of *Granulicatella *spp. in pediatric IE is likely underestimated owing to its frequent misclassification as culture-negative endocarditis. Systematic reporting of dental history, prophylaxis status, and microbiological details in future pediatric IE cases will be essential to better define this rare entity and strengthen preventive strategies.

## References

[1] Vicent L, Luna R, Martínez-Sellés M (2022). Pediatric infective endocarditis: a literature review. J Clin Med.

[2] Eleyan L, Khan AA, Musollari G (2021). Infective endocarditis in paediatric population. Eur J Pediatr.

[3] Holloway V, Jacob G, Hayes N (2021). Challenges in the diagnosis and management of Granulicatella elegans endocarditis in a 9-year-old child. BMJ Case Rep.

[4] Granda-Jiménez MJ, Colin-Ortiz JL, de la Garza EA (2023). Infective endocarditis caused by Granulicatella elegans in a child with patent ductus arteriosus. Pediatr Infect Dis J.

[5] Albano C, Bagarello S, Giordano S (2022). Granulicatella spp., a causative agent of infective endocarditis in children. Pathogens.

[6] Patri S, Agrawal Y (2016). Granulicatella elegans endocarditis: a diagnostic and therapeutic challenge. BMJ Case Rep.

[7] Farid S, Esquer Garrigos Z, Sohail MR (2019). Infective endocarditis due to Granulicatella elegans presenting with musculoskeletal symptoms. BMJ Case Rep.

[8] De Luca M, Amodio D, Chiurchiù S (2013). Granulicatella bacteraemia in children: two cases and review of the literature. BMC Pediatr.

[9] Alhuarrat MA, Garg V, Borkowski P (2024). Epidemiologic and clinical characteristics of marantic endocarditis: a systematic review and meta-analysis of 416 reports. Curr Probl Cardiol.

[10] Delgado V, Ajmone Marsan N, de Waha S (2023). 2023 ESC Guidelines for the management of endocarditis. Eur Heart J.

[11] Li JS, Sexton DJ, Mick N (2000). Proposed modifications to the Duke criteria for the diagnosis of infective endocarditis. Clin Infect Dis.

[12] Téllez A, Ambrosioni J, Llopis J (2018). Epidemiology, clinical features, and outcome of infective endocarditis due to Abiotrophia and Granulicatella species: report of 76 cases. Clin Infect Dis.

[13] Adam EL, Siciliano RF, Gualandro DM (2015). Case series of infective endocarditis caused by Granulicatella species. Int J Infect Dis.

